# Cross-Tissue Analysis Using Machine Learning to Identify Novel Biomarkers for Knee Osteoarthritis

**DOI:** 10.1155/2022/9043300

**Published:** 2022-06-23

**Authors:** Yudong Zhao, Yu Xia, Gaoyan Kuang, Jihui Cao, Fu Shen, Mingshuang Zhu

**Affiliations:** ^1^School of Clinical Medicine, Chengdu University of Traditional Chinese Medicine, 610075, China; ^2^Provincial Key Laboratory of TCM Diagnostics, Hunan University of Chinese Medicine, 410208, China; ^3^Department of Orthopaedics, The First Affiliated Hospital of Hunan University of Chinese Medicine, 410007, China; ^4^Department of Orthopaedics and Traumatology, Changshou District Hospital of Traditional Chinese Medicine, 400000, China; ^5^Department of Orthopaedics, Yong Zhou Hospital of Traditional Chinese Medicine, 425000, China; ^6^Department of Orthopaedics, Hospital of Chengdu University of Traditional Chinese Medicine, 610075, China

## Abstract

**Background:**

Knee osteoarthritis (KOA) is a common degenerative joint disease. In this study, we aimed to identify new biomarkers of KOA to improve the accuracy of diagnosis and treatment.

**Methods:**

GSE98918 and GSE51588 were downloaded from the Gene Expression Omnibus database as training sets, with a total of 74 samples. Gene differences were analyzed by Gene Ontology, Kyoto Encyclopedia of Genes and Genomes pathway, and Disease Ontology enrichment analyses for the differentially expressed genes (DEGs), and GSEA enrichment analysis was carried out for the training gene set. Through least absolute shrinkage and selection operator regression analysis, the support vector machine recursive feature elimination algorithm, and gene expression screening, the range of DEGs was further reduced. Immune infiltration analysis was carried out, and the prediction results of the combined biomarker logistic regression model were verified with GSE55457.

**Results:**

In total, 84 DEGs were identified through differential gene expression analysis. The five biomarkers that were screened further showed significant differences in cartilage, subchondral bone, and synovial tissue. The diagnostic accuracy of the model synthesized using five biomarkers through logistic regression was better than that of a single biomarker and significantly better than that of a single clinical trait.

**Conclusions:**

CX3CR1, SLC7A5, ARL4C, TLR7, and MTHFD2 might be used as novel biomarkers to improve the accuracy of KOA disease diagnosis, monitor disease progression, and improve the efficacy of clinical treatment.

## 1. Introduction

Knee osteoarthritis (KOA) is the most common form of arthritis, it has a significant negative effect on patient quality of life, and it is an important cause of disability in the adult population [1, 2]. As a common degenerative joint disease, the pathogenic factors might be metabolic abnormalities of chondrocytes, subchondral bone, and extracellular matrix caused by the comprehensive effects of heredity, metabolism, biochemistry, and biomechanics [3–5]. Pathological changes are manifested as articular cartilage degeneration and weight-bearing joint cartilage surface disappearance [6], subchondral bone degeneration, osteosclerosis [7], osteophyte formation at the joint edge, and synovial aseptic inflammation [8]. The continuous damage of chronic inflammation and progressive structural changes in the joint tissue lead to the continuous progression of the disease, which finally results in severe pain and loss of joint function [9]. Therefore, it is very important to diagnose and implement intervention measures in the early stages of KOA.

At present, there are many studies reporting the bioinformatics analysis of a single tissue with respect to osteoarthritis (OA). Through immune infiltration analysis of synovial gene data, it was found that *COL3A1* and *MMP9* can be used as potential biomarkers of OA, as confirmed by qRT-PCR and western blot analysis [10]. Wang et al. identified 12 cores of differentially expressed genes (DEGs) through the analysis of gene expression data of the subchondral bone in KOA model mice [11]. An analysis of gene expression data of the KOA cartilage showed that the difference in *BLNK* between the OA and normal cartilage groups was most significant, and silencing BLNK was found to inhibit activation of the NF-*κ*B pathway, thereby inhibiting chondrocyte apoptosis, inflammation, and extracellular matrix degradation [12].

Whereas KOA was once thought to be a disease of articular cartilage alone, it is now widely believed that all joint structures are affected, including cartilage, subchondral bone, and the synovium [13–16]. With a gradual deepening of the understanding of KOA, an increasing number of studies have included multiple tissues in the analysis data. It was found that different periosteal and synovial bone progenitor cells cooperate to form osteophytes in OA [17].

There are several tissues related to osteoarthritis, so the cost of molecular biology experiments is high. In addition, sampling subchondral bone and cartilage is an invasive operation. Therefore, it is necessary to use an accurate prediction model to narrow the range of candidate genes before a biological experiment. This model can improve the experimental efficiency and reduce the cost of trial and error [18]. Some studies have shown that for complex diseases such as OA, multitissue analysis of multiomics methods and longitudinal clinical data are needed to comprehensively understand the disease process and develop effective diagnostics, prognostics, and biotherapies [19]. The synovium and chondrocytes were previously jointly analyzed by single-cell RNA sequencing to explore the pathogenesis of OA in the two tissues [20].

With the rapid development of genome and other sequencing projects, the academic community already has huge public databases, such as Gene Expression Omnibus (GEO), The Cancer Genome Atlas (TCGA), and ArrayExpress. The focus of bioinformatics research is gradually shifting from accumulating data to how to interpret and mine these data. Machine learning methods such as neural networks, decision trees, and support vector machines (SVM) are suitable for dealing with this field with large amounts of data, noise, and a lack of unified theory [21]. Machine learning can obtain hidden clues from a large amount of data. Support vector machine recursive feature elimination (SVM-RFE) algorithm is a powerful feature selection algorithm in machine learning. It was proposed by Guyon et al. in 2002 [22], which is used to find the best variable by deleting the feature vector generated by SVM. When the number of features is large, using SVM-RFE to avoid overfitting is a good choice. Least absolute shrinkage and selection operator (LASSO) regression analysis can shrink the regression coefficients of some variables to zero by imposing constraints on the model parameters, so it retains the advantages of subset shrinkage and minimizes the prediction error [23]. It has been successfully applied in bioinformatics analysis and clinical research related to KOA [24, 25].

In general, machine learning can process complex data through data dimensionality reduction and multiscale modeling to promote further improvements in clinical diagnosis, precision treatment, and health monitoring [26, 27], and this method has been applied to imaging evaluation and gait analysis with KOA [28–30]. The application of machine learning method to the study of chronic joint inflammation similar to KOA has also been successful [31]. Jamshidi et al. [32] proposed the use of a machine learning method to mine KOA data and improve clinical decision-making and precision medicine, but there is no literature on its practical application. This study is aimed at analyzing and verifying the published KOA multitissue gene expression data using machine learning and bioinformatics methods, looking for potential diagnostic markers and therapeutic targets.

## 2. Materials and Methods

### 2.1. Data Sources

The series of matrix files used in this study were all from the public data published in the Gene Expression Omnibus (GEO) database (http://www.ncbi.nlm.nih.gov/geo/), obtained by searching for “osteoarthritis “knee osteoarthritis” “KOA”. GSE98918 and GSE51588 were used as training sets, and GSE55457 was used as the validation set. Basic information of the three gene matrices is listed in Table 1. Clinical data from the validation set were extracted for further analysis (Supplementary Table 1).

### 2.2. Differential Expression Analysis

The “Sva” package in R was used to merge the two training sets of chips, and the merged data were normalized to eliminate the batch effect. Using the “limma” package in R to extract the differential genes, gene screening difference conditions were set to ∣logFC value | >1 and FDR values < 0.05 [33].

### 2.3. Enrichment Analysis

Gene Ontology (GO), Kyoto Encyclopedia of Genes and Genomes (KEGG) pathway, and Disease Ontology (DO) enrichment analyses were performed on the screened DEGs using the “clusterProfiler” package in R. The screening threshold was set at *p* < 0.05 to meet the statistical significance requirement [34]. Enrichment analysis of GSEA was performed using “c2.cp.kegg.v7.4.symbols.gmt” and “c5.go.v7.4.symbols.gmt” in the Molecular Signatures Database (MSigDB; http://www.gsea-msigdb.org/gsea/msigdb) as the reference gene sets.

### 2.4. Screening Diagnostic Biomarkers

The least absolute shrinkage and selection operator (LASSO) regression analysis of DEGs was carried out with the “glmnet” package in R to further reduce the range of candidate diagnostic genes, and the number of folds is set to the default value of 10. According to the support vector machine (SVM) assessment, the variables most related to the research results were selected, and the best candidate diagnostic gene combination was selected by ranking the correlation strength [35]. The intersecting genes from the two algorithms were obtained as candidate biomarkers and finally determined through gene expression screening.

### 2.5. Validation of Diagnostic Biomarkers

Identified biomarkers were used to generate the receiver operating characteristic (ROC) curve, judge the predictive effect of biomarkers according to the area under the ROC curve (AUC), and use the ROC curve to judge the diagnostic accuracy in GSE55457. At the same time, we used the identified biomarkers to fit the diagnostic model by logistic regression and compared the predictive effect of the diagnostic model with the ROC curve of clinical traits in the validation set, to judge the diagnostic effect of identified biomarkers.

### 2.6. Immune Infiltration Analysis

Immune cell infiltration refers to the migration of immune cells from the blood to the tissue, and the proportion of immune cells is calculated from the gene expression in tissue samples [36]. The effectiveness of the Cell-type Identification by Estimating Relative Subsets of RNA Transcripts (CIBERSORT) deconvolution algorithm has been verified by flow cytometry [37]. The CIBERSORT deconvolution algorithm was used to perform immune infiltration analysis on the training set to evaluate the differences in the proportions of immune cells between KOA and normal samples. A correlation test was performed on identified biomarkers and immune cells, scatter and lollipop diagrams were generated, and the threshold was set to a correlation coefficient *p* value of gene expression and immune cells < 0.05.

## 3. Results

### 3.1. Differential Expression Analysis

**Figure 1:**
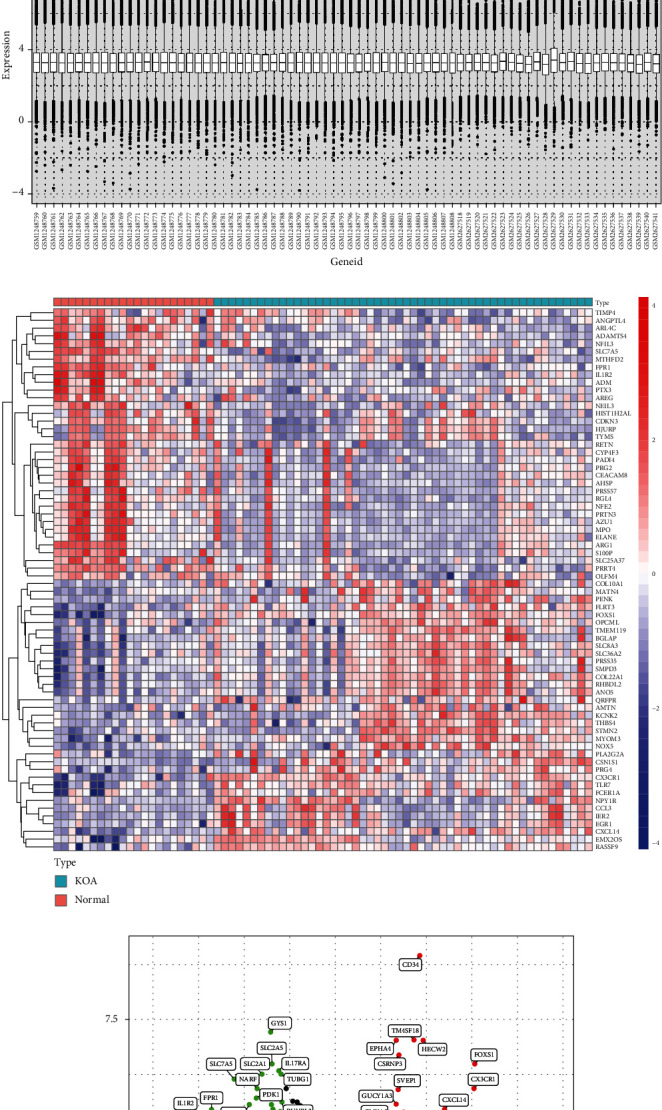
Box plot of merged gene expression (a), normalized gene expression (b), gene heat map (c), and volcano map (d) of differential expression between knee osteoarthritis (KOA) tissue and normal samples.

Box plot of merged gene expression is shown in Figure 1(a), and normalized gene expressions were drawn in Figure 1(b). The box plot showed that the expression data of different organizations had been batch normalized. In total, 84 DEGs (47 significantly downregulated and 37 significantly upregulated) were identified between OA and normal samples (see Supplementary Table 2). All DEGs were used to generate a heat map (Figure 1(c)) and a volcano map (Figure 1(d)).

### 3.2. Enrichment Analysis of DEGs

**Figure 2:**
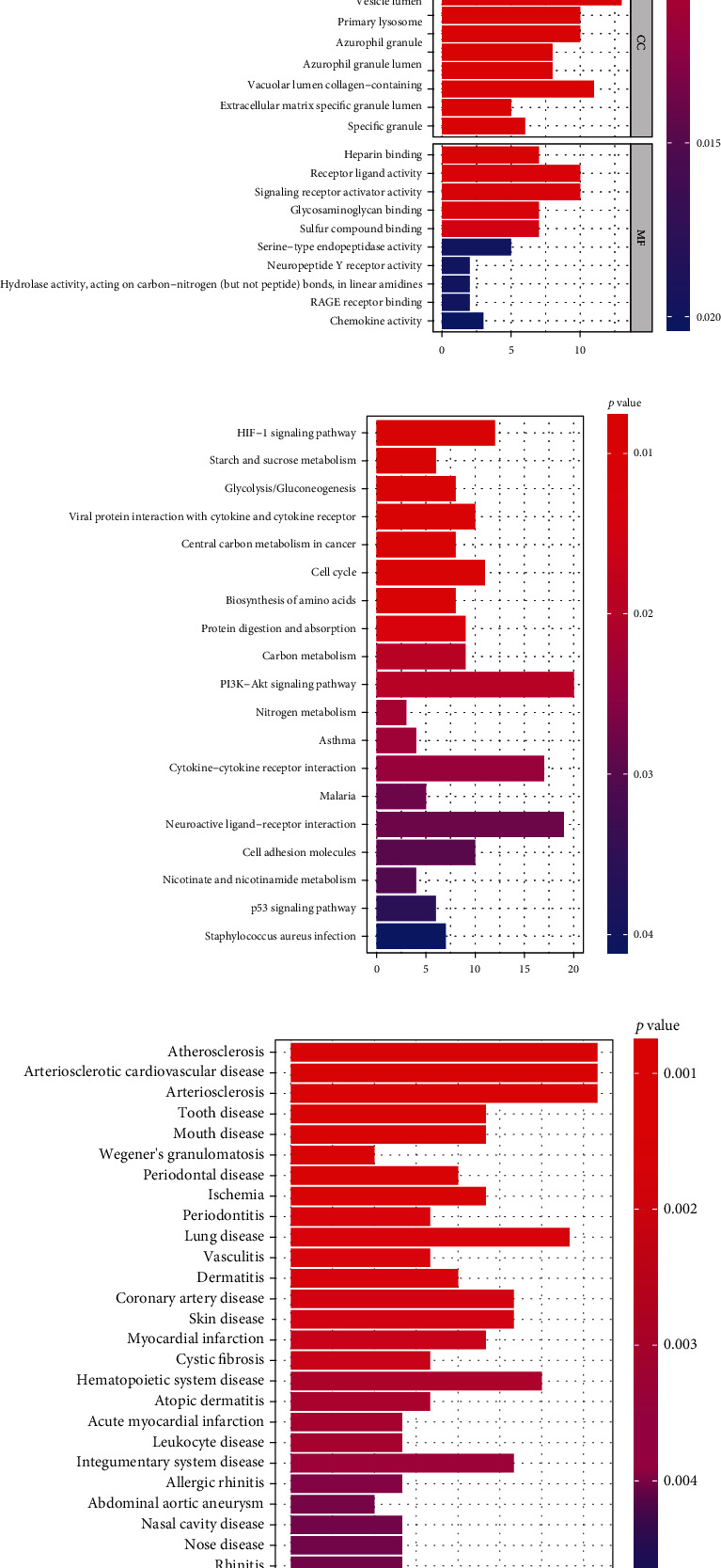
Analysis results of differentially expressed genes (DEGs) for Gene Ontology (GO) functional enrichment (a), Kyoto Encyclopedia of Genes and Genomes (KEGG) pathway enrichment (b), and Disease Ontology (DO) enrichment (c) in knee osteoarthritis (KOA) and normal samples.

GO functional enrichment analysis (Figure 2(a)) showed that DEGs were enriched in neutrophil degranulation, neutrophil activation involved in immune response, collagen-containing extracellular matrix, primary lysosome, and vesicle lumen; KEGG pathway analysis (Figure 2(b)) showed that the DEGs were concentrated in the HIF-1 signaling pathway, PI3K-AKT signaling pathway, and cell cycle, whereas DO enrichment analysis (Figure 2(c)) showed that DEGs were significantly expressed in cardiovascular diseases, periodontal disease, and OA.

### 3.3. GSEA

**Figure 3:**
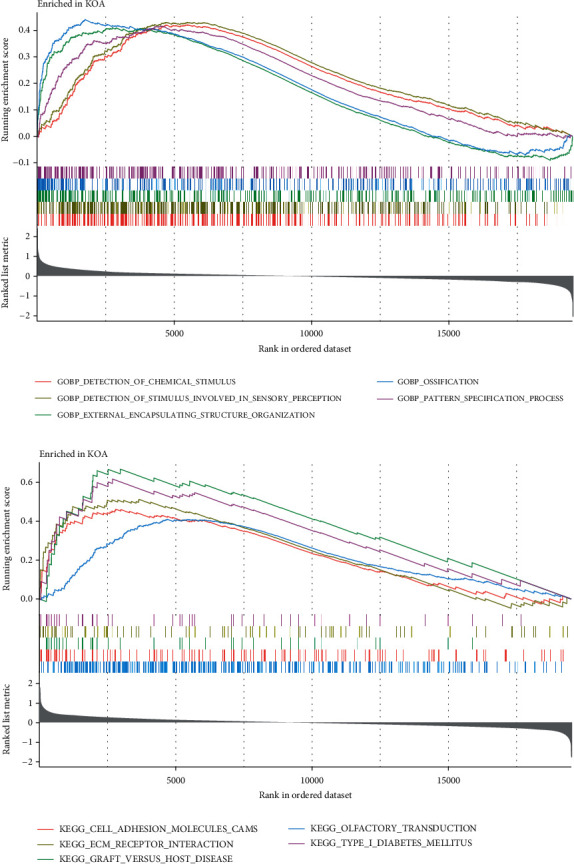
Gene Ontology (GO) functional (a) and Kyoto Encyclopedia of Genes and Genomes (KEGG) pathway (b) analyses of the top five enrichment levels in the gene set enrichment analysis (GSEA).

GSEA is aimed at analyzing the ranked list of all available genes without a threshold and can consider the differences between KOA and normal gene sets from a more comprehensive perspective [38]. GSEA was carried out on KOA sample gene sets, and the gene functions of the top five enrichment degrees were (Figure 3(a)) as follows: detection of chemical stimulus, detection of stimulus involved in sensory perception, external encapsulating structure organization, ossification, and pattern specification process; the first five enriched KEGG pathways were (Figure 3(b)) as follows: cell adhesion molecule CAMs, ECM receptor interaction, graft-versus-host disease, olfactory transduction, and type I diabetes mellitus.

### 3.4. Identification of Diagnostic Biomarkers

**Figure 4:**
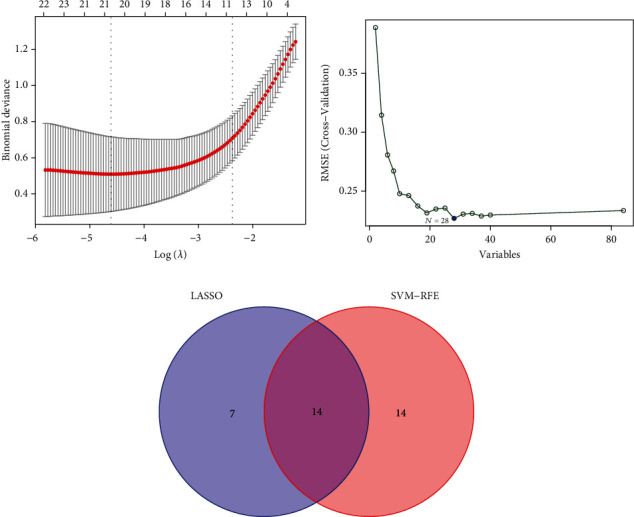
In the least absolute shrinkage and selection operator (LASSO) regression model, when adjusted to the optimal lambda, the number of genes with a nonzero coefficient was 21 (a). The results of support vector machine recursive feature elimination (SVM-RFE) algorithm showed that when the number of genes was 28, the cross-validation error was smallest (b). The intersecting genes of the two methods included 14 genes (c).

By LASSO regression analysis of DEGs, 21 candidate biomarkers were obtained (Figure 4(a)), and 28 total candidate biomarkers were screened using the support vector machine recursive feature elimination (SVM-RFE) algorithm (Figure 4(b)). In total, 14 intersecting genes of the two algorithms were considered and used to generate the Venn diagram (Figure 4(c)).

**Figure 5:**
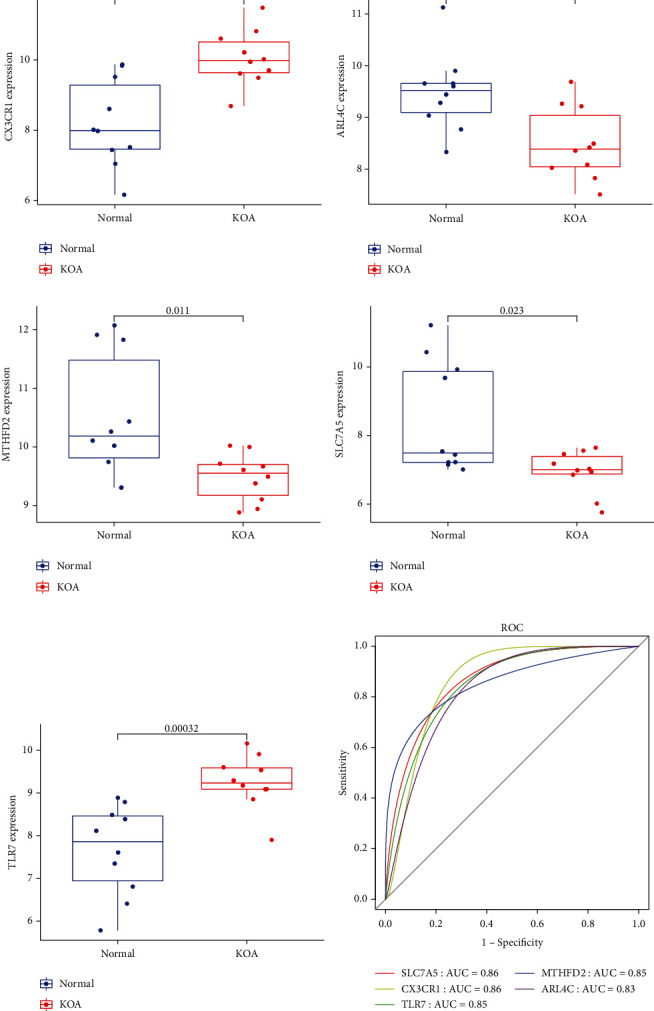
Expression of diagnostic biomarkers in the validation dataset: *CX3CR1* (a), *ARL4C* (b), *MTHFD2* (c), *SLC7A5* (d), and *TLR7* (e). Receiver operator characteristic (ROC) curve of five genes in training set (f).

The expression of 14 intersecting genes was observed in the training dataset. The box plot (Figures 5(a)–5(e)) showed that there were significant differences in the expression of five genes (*p* < 0.05). The ROC curves of *CX3CR1*, *SLC7A5*, *ARL4C*, *TLR7*, and *MTHFD2* in the training set (Figure 5(f)) showed that the AUC values were greater than 0.8, which symbolizes a good predictive effect.

### 3.5. Verification of Identified Biomarkers

**Figure 6:**
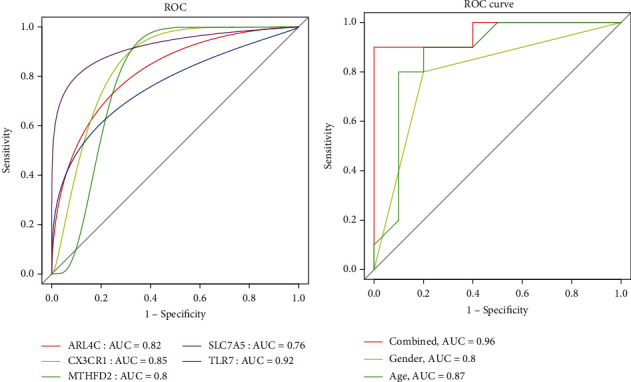
Receiver operator characteristic (ROC) curves of five diagnostic biomarkers in the validation set (a). ROC curves of combined biomarker model and clinical data (b).

According to the ROC curves of the five identified biomarkers in the GSE55457 gene set (Figure 6(a)), all AUC values were greater than 0.8 and had good predictive ability, with an AUC of 0.83 for *ARL4C*, 0.90 for *CX3CR1*, 0.83 for *MTHFD2*, 0.80 for *SLC7A5*, and 0.94 for *TLR7*. At the same time, we found that the diagnostic effect of the combined model was better than that with the clinical data (Figure 6(b)), with an AUC of 0.96 in the combined biomarker model, an AUC of 0.80 with the sex-based model, and an AUC of 0.87 for the age model.

### 3.6. Immune Infiltration Analysis

**Figure 7:**
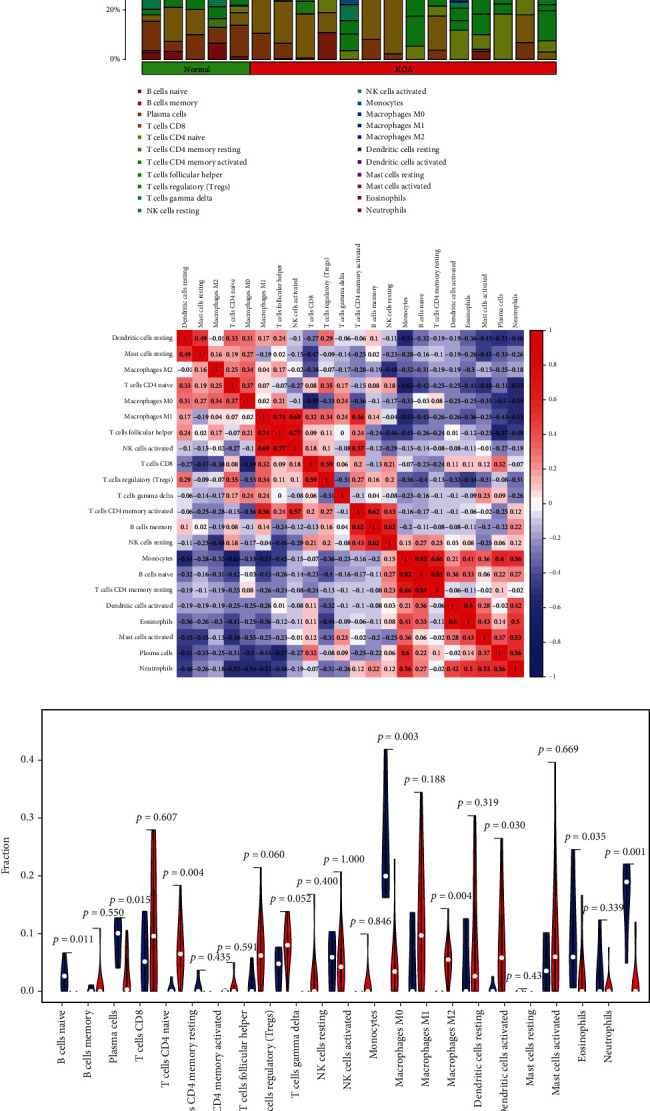
The immune infiltration histogram (a), correlation heat map (b), and violin plot (c) of immune cells in the training set.

The immune infiltration histogram (Figure 7(a)) and correlation heat map (Figure 7(b)) of the training set showed that CD4 memory T cell resting was positively correlated with naïve B cells, with a correlation coefficient of 0.84, whereas monocytes were negatively correlated with naïve CD4 T cells, with a correlation coefficient of −0.63.

The violin plot (Figure 7(c)) showed differences in the proportions of eight types of immune cells between normal samples and KOA samples; compared with those in normal samples, the proportions of naïve B cells, plasma cells, monocytes, resting dendritic cells, activate mast cells, and neutrophils in OA samples were lower, and the proportions of naive CD4 T cells and M1 macrophages in OA samples were higher.

### 3.7. Correlation between Identified Biomarkers and Immune Cells

**Figure 8:**
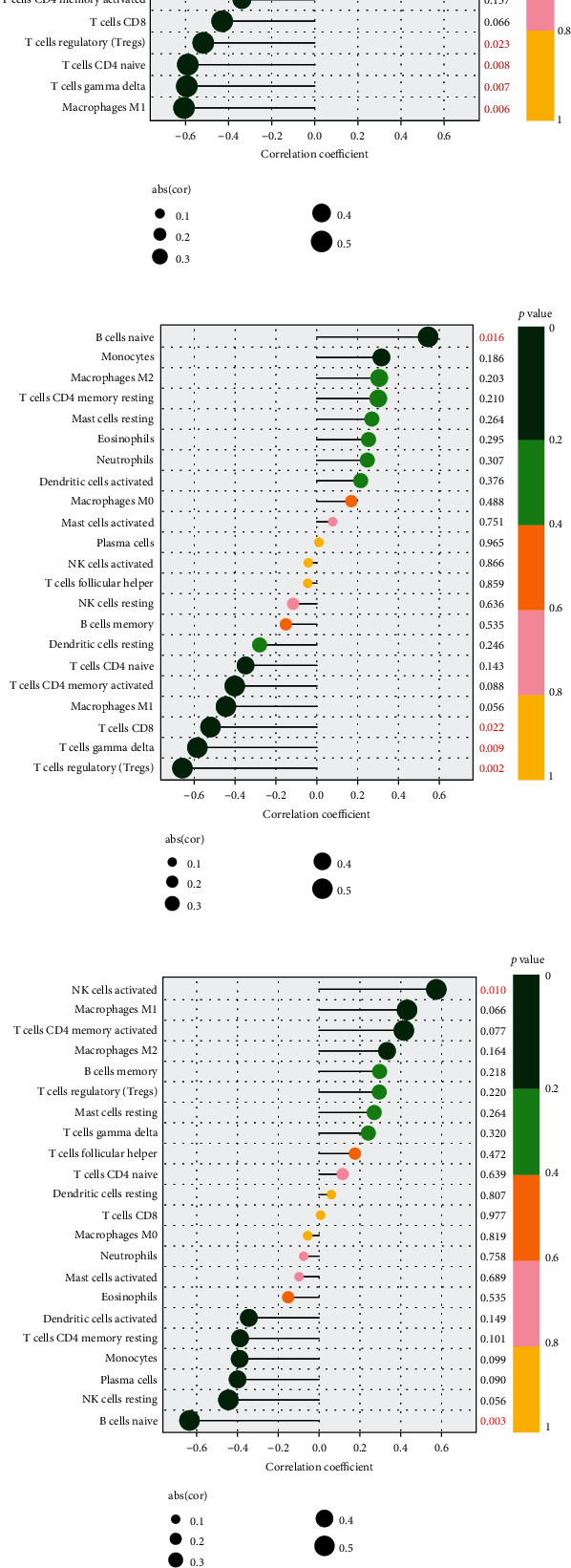
Lollipop diagram of immune cells and five diagnostic genes: *CX3CR1* (a), *SLC7A5* (b), *ARL4C* (c), *TLR7* (d), and *MTHFD2* (e).

The lollipop diagram showed that with *p* values less than 0.05 as the screening threshold, there were two types of immune cells related to *CX3CR1* expression (Figure 8(a)), gamma delta T cells and M1 macrophages. There were six types of immune cells associated with *SLC7A5* (Figure 8(b)), naïve B cells, M1 macrophages, gamma delta T cells, naive CD4 T cells, regulatory T cells (Tregs), and monocytes. There were four types of immune cells associated with *ARL4C* (Figure 8(c)), regulatory T cells (Tregs), gamma delta T cells, naïve B cells, and CD8 T cells. There were two types of immune cells associated with *TLR7* (Figure 8(d)), naïve B cells and activated NK cells. Immune cells associated with *MTHFD2* included CD8 T cells (Figure 8(e)). The correlation tendency between identified biomarkers and immune cells is shown by a scatter diagram (Supplementary Figures 1–15).

## 4. Discussion

Based on the gene expression data published in the GEO database, this study involved performing machine learning and bioinformatics analysis of the gene expression data related to KOA from the perspective of multiple tissue combinations. This was performed to find the common diagnostic biomarkers and therapeutic targets in multiple tissues affected by KOA. In total, five biomarkers showing significant differences in the cartilage and subchondral bone tissue were obtained. These five genes were combined into a diagnostic gene model using logistic regression. The diagnostic accuracy of the model was better than that of any single gene in the validation set, and the diagnostic accuracy was significantly better than that of a single clinical trait. These results suggest that this method can potentially be used as a new basis for more accurate disease diagnosis, monitoring disease progression, and reflecting clinical efficacy.

MRI can often be used to detect bone marrow lesions and synovial hypertrophy in the early stages of KOA, both of which can precede cartilage damage [39]. The synovium, subchondral bone, and cartilage are hotspots in the study of KOA diseases. Most studies on KOA have focused on analyzing data from a single tissue sample. As a result, there are very few research articles that report the use of data from across several tissues. KOA has an effect on the cartilage, subchondral bone, synovium, and even monocytes in the blood. Therefore, there are some limitations to DEG analysis from a single tissue.

Neutrophil responses to immunity and vesicles, lysosomes, and the collagen-containing extracellular matrix were highly enriched in the GO analysis of common DEGs between the cartilage and subchondral bone microarrays, and these functions are related to the destruction of the cartilage and subchondral bone in KOA. It was found that in contrast to the conventional cognitive neutrophil-mediated proinflammatory process, neutrophil-derived microbubbles can inhibit the TNF-*α*-stimulated secretion of broad-spectrum proinflammatory cytokines [40]. Several studies [41–43] have shown that neutrophil elastase is deeply involved in cartilage damage in OA and functions by activating MMP13 and the caspase signaling pathway. Cell experiments showed that subchondral bone mesenchymal stromal cells can produce external vesicles supporting chondrocyte viability and chondrogenic gene expression and that they contain microRNAs related to chondrogenesis support [44]. By observing the extracellular vesicles released by immune cells in the plasma and synovial fluid, it is speculated that extracellular vesicles can be used as a marker to reflect KOA joint inflammation and disease severity [45]. These studies have shown that neutrophils and outer vesicles play a role in intercellular communication and participate in immunity in the microenvironment of knee joints [46], and many functions with the highest enrichment degree in the GO enrichment analysis in this study are related to this [47, 48].

KEGG pathway enrichment analysis showed that DEGs were widely involved in the HIF-1 signaling pathway, PI3K/AKT signaling pathway, and cell cycle. HIF-1*α* can damage the cartilage tissue by affecting glycolytic metabolism in chondrocytes [49]; one experiment found that compared with that in healthy controls, the level of HIF-1*α* in the human KOA group is enhanced [50]. Western blot experiments showed that activating PI3K/AKT signaling in OA model mice can regulate cartilage degradation in vivo [51]. KOA is a degenerative joint disease, and an increasing number of studies on aging cells and apoptosis have shown that the cell cycle is closely related to KOA [52–54].

The screened DEGs were significantly expressed in cardiovascular diseases, periodontal-related inflammation, and OA. With increasing research on the association between diseases, the relationship between cardiovascular diseases and KOA has been disclosed [55]. Published meta-analysis and cross-sectional studies have shown that cardiovascular diseases can affect the progression of OA [56, 57]. Other studies have shown that periodontitis is associated with the presence and severity of KOA [58]. It has been concluded that periodontitis is at least partly involved in the pathogenesis of OA, especially in patients with type 2 diabetes [59].

Among the five identified biomarkers, *CX3CR1* has been confirmed to regulate the activity of the NF-*κ*B pathway, and it can promote the production of MMP3 in OA synovial fibroblasts [60, 61]. CX3CR1 can induce immune cells to penetrate blood vessels and continuously enter inflammatory sites [62], and it can also be associated with the Wnt/*β*-catenin signaling pathway to regulate chondrocyte proliferation and apoptosis in KOA [63]. Clinical trials have shown that CX3CR1 can reflect the severity of symptoms in patients with KOA [64]. Case control studies have shown that the allele frequency of TLR-7 rs179010 is significantly different between KOA cases and healthy controls [65]. Carrion et al. [66] confirmed that TLR-7 is highly expressed in KOA fibroblast-like synovial cells. It was found that extracellular miR-21, released from synovial tissue, mediates knee pain in surgical OA model rats through TLR7 activation, and a TLR7 antagonist could exert lasting analgesic effects on KOA [67]. MTHFD2 is a mitochondrial single-carbon folate metabolic enzyme. Most of the current studies on MTHFD2 are related to tumors and cardiovascular diseases, and there are no reports related to KOA. However, MTHFD2-dependent glycine is very important for angiogenesis [68], and the hypothesis of the involvement of angiogenesis in KOA has been confirmed by experiments [16]. We speculate that the correlation between KOA and MTHFD2 expression might be caused by angiogenesis and metabolic mechanisms, which needs to be further confirmed by subsequent experiments. SLC7A5, as an amino acid transporter, participates in cell invasion and regulates the protein levels of MMP3 and MMP13 through mTOR signaling in rheumatoid arthritis fibroblast-like synovial cells [69]. Alles et al. [70] linked SLC7A5 expression and downstream signaling pathways to pain. SLC7A5 mediates IL-1 production by monocytes and macrophages, thus participating in chronic inflammatory diseases [71]. In vitro and in vivo experiments showed that the knockdown of ARL4C inhibits the osteogenesis of human adipose stem cells [72]. ARL7, together with ARL4 and ARL6, forms a small subfamily, and they are related to each other through a common C-terminus, thus inducing nuclear localization. As a direct target of the liver X receptor, ARL7 plays a synergistic role in the coordinated regulation of macrophage lipid metabolism and inflammatory gene programs [73].

Immune cell infiltration has been proven to play an important role in the study of KOA. Histological analysis and RNAseq data indicate that M1 macrophages are the important source of joint inflammation [74]. Faust et al. [75] found that CD4+ T cells contribute to the expression of IL-17 and promote joint degeneration in this way. Whitmire et al. [76] provided direct evidence of the requirement of B cells for the establishment of memory CD4 T cells. A test of peripheral blood showed that the frequency of B cells in KOA patients is lower than that in a healthy control group [77]. Meta-analysis showed that the level of serum monocyte chemoattractant protein-1 in patients with OA was significantly higher than that in the control group; however, this difference was not significant in synovial fluid and cartilage [78]. Monocyte-derived cells were confirmed to promote cartilage repair in OA [79]. Wang et al. [80] found that mast cell-derived tryptase induces inflammation, chondrocyte apoptosis, and cartilage decomposition. Chakraborty et al. [81] showed that mechanical stiffness facilitates dendritic cell proinflammatory functions. The large amount of evidence mentioned previously verifies the accuracy of our current research results to a certain extent. Several specific types of immune cells play a vital role in KOA, which can be used as a direction for further research.

Both SVM-RFE and lasso regression selected in this paper are classic supervised learning algorithms, but they are not the only methods for screening diagnostic markers. With the communication between the field of computational intelligence (CI) algorithms and biomedicine, more cutting-edge swarm intelligence (SI) optimization algorithms are used to improve the efficiency of diagnostic models in the medical field [82]. For example, the combination of Harris hawks optimization (HHO), cuckoo search (CS), and SVM method for drug design and discovery has achieved good results [83]. One study used an SVM model optimized by slime mould algorithm (SMA) in combination with random forest method to identify the severity of COVID-19 patients [84], and another study used colony predation algorithm (CPA) in combination with kernel extreme learning machine (KELM) to analyze the biochemical indicators and prognosis of COVID-19 patients [85], both showing high prediction accuracy and stability. Applying monarch butterfly optimization (MBO) to medical image recognition can significantly reduce mean square error (MSE), and the efficiency is better than the existing traditional technology [86]. Elephant herding optimization (EHO) has achieved 90.6% and 88% accuracy in MRI image discrimination and PIMA diabetic dataset classification, respectively [87]. The Runge-Kutta optimizer (RUN) algorithm has a faster convergence speed, higher convergence accuracy, and better optimization ability than similar SI optimization algorithms [88]. Other SI optimization methods with potential for biomedical applications include earthworm optimization algorithm (EWA) [89], moth search (MS) algorithm [90], and hunter games search (HGS) [91]. Horizontal comparisons show that there are differences in the performance of the same SI algorithm in different disease datasets. In actual use, appropriate SI optimization algorithms should be selected according to the characteristics and requirements of the datasets to be processed [92]. Considering the demand for processing a large amount of data in the medical field, this kind of interdisciplinary application has great potential. At the same time, it puts forward higher requirements for biomedical researchers and algorithm engineers, which is worthy of further research.

The biggest challenge our team encountered in this study is how to reduce the bias and error of machine learning algorithms on gene dataset analysis. Firstly, is the bias caused by the difference in input samples? At present, gene expression matrices of osteoarthritis in public databases such as GEO comes from various tissues: cartilage, subchondral bone, and synovium, even blood monocytes. Due to the influence of inflammatory substances and immune response, there are different degrees of gene expression between normal and KOA samples in these tissues. Therefore, the first problem to be solved is to choose which tissue gene dataset to analyze. Our team spent several months trying to combine and analyze all gene datasets from a single tissue source but finally failed. The reason is that these public gene expression data come from different research teams, due to different sequencing methods and sampling methods, direct combination and comparison will produce huge errors. Second, there are few KOA-related gene sequencing datasets uploaded to the public database, and the low amount of data in a single tissue dataset will also reduce the accuracy of the analysis results.

To meet these challenges, we turned to focus on periarticular tissue (the cartilage, subchondral bone, and synovium) for combined analysis. By analyzing the differential genes of each group's gene dataset, respectively, and then conducting batch normalization, we reduced the intergroup error and increased the sample size by merging the gene dataset from different tissues, At the same time, the accuracy of analysis is improved through verification, so that the common diagnostic biomarkers existing in different periarticular tissues can be obtained.

This study also has some limitations. Firstly, this study obtained the common diagnostic biomarkers of multiple tissues through combined analysis, which will also reduce the possibility of obtaining tissue-specific biomarkers that only exist in certain tissue. Secondly, because the gene datasets come from the public database, we cannot obtain more clinical information for in-depth correlation analysis. Finally, due to the lack of experimental conditions, we failed to further verify the predicted diagnostic markers at the molecular biological level. Follow-up animal experiments and large sample prospective studies are needed to confirm the results of this study.

## 5. Conclusions

In this study, bioinformatics methods and machine learning algorithms were used to analyze multitissue gene expression data. *CX3CR1*, *SLC7A5*, *ARL4C*, *TLR7*, and *MTHFD2* were identified as diagnostic markers for KOA, and they have great potential to comprise a new diagnostic and therapeutic target. Although most of the analysis results have been confirmed to a certain extent by published experimental studies, the speculation on some unknown mechanisms in this article still needs further experiments to confirm the influence on KOA.

## Figures and Tables

**Table 1:** Knee osteoarthritis (KOA) gene expression dataset in the Gene Expression Omnibus database.

GEO ID	Platform	Source	Normal	OA
GSE98918	GPL20844	Meniscus	12	12
GSE51588	GPL13497	Subchondral	10	40
GSE55457	GPL96	Bone Synovium	10	10

## Data Availability

The GEO database is a public database. Patients involved in the database were subjected to ethical approval. Users can download relevant data free for research and to publish relevant articles. Our study is based on open-source data, and thus, there are no ethical issues or conflicts of interest. The data and materials used in the current study are available from the corresponding author upon reasonable request.

## Supplementary Materials

Supplementary Materials Supplementary Table 1: clinical data from the validation set (GSE55457). Supplementary Table 2: DEGs identified from KOA and normal samples. Supplementary Figure 1-15: scatter plot of correlation trend between identified biomarkers and immune cells.
