# Supplementary material for: Cross-Tissue Analysis Using Machine Learning to Identify Novel Biomarkers for Knee Osteoarthritis
Source: Comput Math Methods Med. 2022 Jun 23;2022:9043300. doi: 10.1155/2022/9043300 (PMC9246600; doi:10.1155/2022/9043300)
Supplement: Supplementary Materials — Supplementary Table 1: clinical data from the validation set (GSE55457). Supplementary Table 2: DEGs identified from KOA and normal samples. Supplementary Figure 1-15: scatter plot of correlation trend between identified biomarkers and immune cells. [file 9043300.f1.docx]

**Supplementary Materials**

Supplementary Table 1: Clinical data from the validation set (GSE55457)

| ID | Gender | Age(y) | Status |
| --- | --- | --- | --- |
| GSM1337304 | Male | 61 | Normal |
| GSM1337305 | Male | 64 | Normal |
| GSM1337306 | Female | 78 | Normal |
| GSM1337307 | Male | 65 | Normal |
| GSM1337308 | Male | 53 | Normal |
| GSM1337309 | Male | 68 | Normal |
| GSM1337310 | Female | 29 | Normal |
| GSM1337311 | Male | 17 | Normal |
| GSM1337312 | Male | 39 | Normal |
| GSM1337313 | Male | 36 | Normal |
| GSM1337327 | Female | 77 | KOA |
| GSM1337328 | Female | 71 | KOA |
| GSM1337329 | Female | 76 | KOA |
| GSM1337330 | Female | 61 | KOA |
| GSM1337331 | Female | 75 | KOA |
| GSM1337332 | Male | 78 | KOA |
| GSM1337333 | Male | 69 | KOA |
| GSM1337334 | Female | 71 | KOA |
| GSM1337335 | Female | 80 | KOA |
| GSM1337336 | Female | 66 | KOA |

Supplementary Table 2. DEGs identified from KOA and normal samples

| Gene | logFC | P. Value | FDR |
| --- | --- | --- | --- |
| *FOXS1* | 1.531856 | 1.25E-10 | 3.25E-07 |
| *SLC7A5* | -1.10941 | 4.45E-10 | 7.23E-07 |
| *CX3CR1* | 1.523444 | 8.78E-10 | 1.19E-06 |
| *CXCL14* | 1.200293 | 5.00E-09 | 3.50E-06 |
| *FPR1* | -1.35812 | 5.31E-09 | 3.57E-06 |
| *IL1R2* | -1.53165 | 7.44E-09 | 4.18E-06 |
| *COL22A1* | 1.40423 | 1.07E-08 | 5.67E-06 |
| *PRRT4* | -1.39246 | 1.25E-08 | 6.28E-06 |
| *TLR7* | 1.109492 | 1.69E-08 | 6.87E-06 |
| *MYOM3* | 1.034145 | 1.81E-08 | 7.20E-06 |
| *MTHFD2* | -1.06524 | 5.84E-08 | 1.58E-05 |
| *SLC25A37* | -1.07612 | 6.02E-08 | 1.59E-05 |
| *PIM1* | -1.00932 | 6.59E-08 | 1.71E-05 |
| *FLRT3* | 1.372729 | 1.07E-07 | 2.51E-05 |
| *ARL4C* | -1.06838 | 1.10E-07 | 2.56E-05 |
| *ADAMTS4* | -1.27435 | 1.34E-07 | 2.98E-05 |
| *EREG* | -1.05559 | 1.95E-07 | 3.96E-05 |
| *PRTN3* | -1.86047 | 2.75E-07 | 4.91E-05 |
| *RASSF9* | 1.451764 | 3.94E-07 | 6.40E-05 |
| *RGL4* | -1.32678 | 4.38E-07 | 6.95E-05 |
| *NFIL3* | -1.213 | 6.82E-07 | 9.25E-05 |
| *NMNAT2* | 1.03232 | 1.02E-06 | 0.000121 |
| *GADD45A* | -1.0173 | 1.50E-06 | 0.000156 |
| *NEIL3* | -1.0868 | 2.47E-06 | 0.000221 |
| *FCER1A* | 1.149859 | 4.17E-06 | 0.000316 |
| *ARG1* | -1.25087 | 5.72E-06 | 0.000385 |
| *CSN1S1* | 1.171217 | 6.09E-06 | 0.000398 |
| *SLC36A2* | 1.102447 | 7.03E-06 | 0.000433 |
| *RHBDL2* | 1.123034 | 9.79E-06 | 0.000531 |
| *SLC8A3* | 1.085085 | 9.91E-06 | 0.000533 |
| *AZU1* | -1.81377 | 1.05E-05 | 0.000556 |
| *PRG4* | 1.78568 | 1.19E-05 | 0.000602 |
| *ANO5* | 1.245685 | 1.34E-05 | 0.000643 |
| *ANGPTL4* | -1.14464 | 1.60E-05 | 0.000725 |
| *PRSS57* | -1.46355 | 1.72E-05 | 0.00076 |
| *CDKN3* | -1.11939 | 1.80E-05 | 0.000784 |
| *IER2* | 1.046503 | 2.14E-05 | 0.000881 |
| *UBE2C* | -1.01753 | 2.42E-05 | 0.000965 |
| *ADM* | -1.26736 | 2.46E-05 | 0.000973 |
| *KCNK2* | 1.471905 | 2.82E-05 | 0.001083 |
| *SMPD3* | 1.658266 | 2.98E-05 | 0.001118 |
| *ELANE* | -1.51857 | 3.41E-05 | 0.001182 |
| *PLA2G2A* | 1.360031 | 3.83E-05 | 0.001285 |
| *PLIN2* | -1.05535 | 4.09E-05 | 0.001326 |
| *GNG4* | 1.019828 | 4.16E-05 | 0.001342 |
| *PENK* | 1.297914 | 4.78E-05 | 0.001467 |
| *BGLAP* | 1.242866 | 4.81E-05 | 0.001471 |
| *HIST1H2AL* | -1.09574 | 4.92E-05 | 0.001494 |
| *PRG2* | -1.13453 | 6.32E-05 | 0.001746 |
| *EMX2OS* | 1.218388 | 7.97E-05 | 0.002043 |
| *MPO* | -2.04288 | 8.65E-05 | 0.002158 |
| *PADI4* | -1.17161 | 9.47E-05 | 0.002303 |
| *NOX5* | 1.170448 | 0.0001 | 0.002416 |
| *S100P* | -1.24203 | 0.00012 | 0.002653 |
| *PTX3* | -1.1834 | 0.00014 | 0.002946 |
| *STMN2* | 2.394991 | 0.00014 | 0.00296 |
| *C10orf10* | -1.06215 | 0.00017 | 0.003484 |
| *HJURP* | -1.13387 | 0.00028 | 0.004791 |
| *AREG* | -1.16278 | 0.00029 | 0.004957 |
| *COL10A1* | 1.234687 | 0.00032 | 0.005228 |
| *TMEM119* | 1.081128 | 0.00043 | 0.006422 |
| *CCNB2* | -1.03999 | 0.00047 | 0.006849 |
| *NFE2* | -1.09489 | 0.00047 | 0.006861 |
| *CCL3* | 1.499893 | 0.00048 | 0.006887 |
| *RNASE2* | -1.03433 | 0.00054 | 0.007449 |
| *RETN* | -1.14889 | 0.00056 | 0.00762 |
| *HIST1H3B* | -1.01641 | 0.00057 | 0.007721 |
| *PRSS35* | 1.239141 | 0.00061 | 0.008096 |
| *OPCML* | 1.102694 | 0.00068 | 0.00868 |
| *CEACAM8* | -1.31774 | 0.00074 | 0.009156 |
| *CYP4F3* | -1.08083 | 0.00078 | 0.009477 |
| *PF4* | -1.00457 | 0.00085 | 0.00996 |
| *MATN4* | 1.170109 | 0.00092 | 0.01051 |
| *THBS4* | 1.127989 | 0.00102 | 0.011254 |
| *TYMS* | -1.09076 | 0.00108 | 0.011742 |
| *AHSP* | -1.13501 | 0.0012 | 0.012573 |
| *TIMP4* | -1.17038 | 0.00131 | 0.013326 |
| *EGR1* | 1.222435 | 0.0014 | 0.013866 |
| *NPY1R* | 1.248852 | 0.00141 | 0.013918 |
| *HIST1H2AI* | -1.05334 | 0.00164 | 0.015351 |
| *QRFPR* | 1.054796 | 0.00197 | 0.017508 |
| *RHAG* | -1.06426 | 0.00265 | 0.021498 |
| *AMTN* | 1.041753 | 0.00407 | 0.02846 |
| *OLFM4* | -1.29585 | 0.00472 | 0.031189 |

Supplementary figure 1-15: Scatter plot of correlation trend between identified biomarkers and immune cells


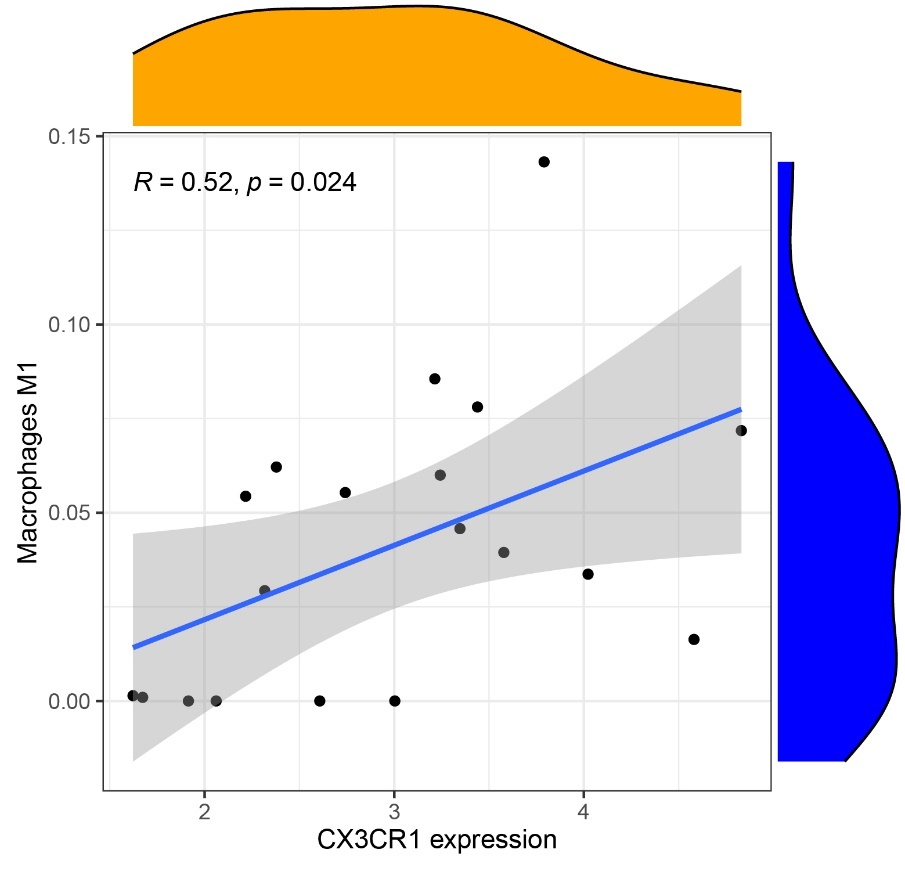


Supplementary figure 1: Correlation trend between *CX3CR1* and M1 macrophages


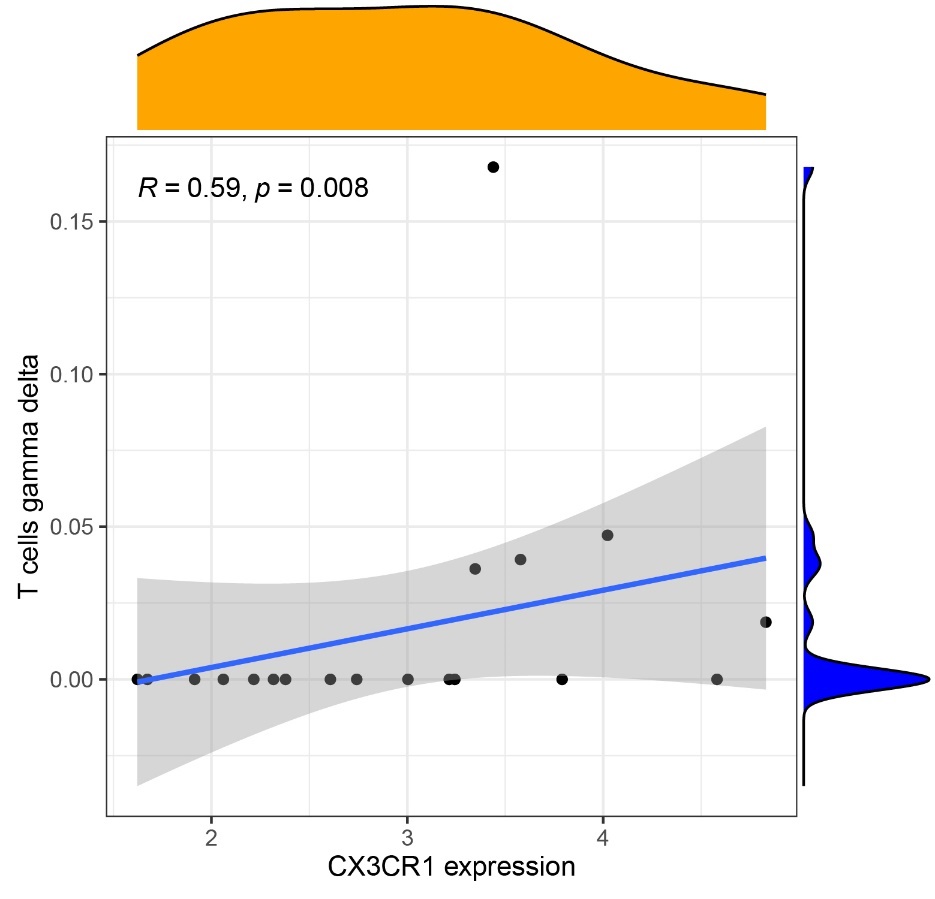


Supplementary figure 2: Correlation trend between *CX3CR1* and gamma delta T cells


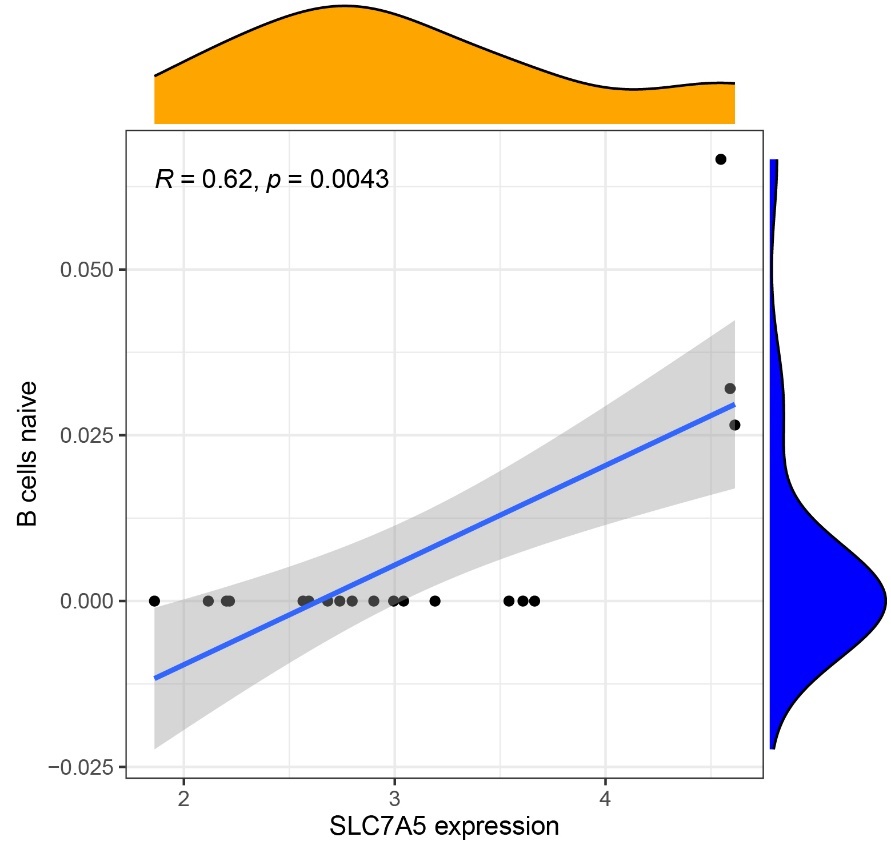


Supplementary figure 3: Correlation trend between *SLC7A5* and naïve B cells


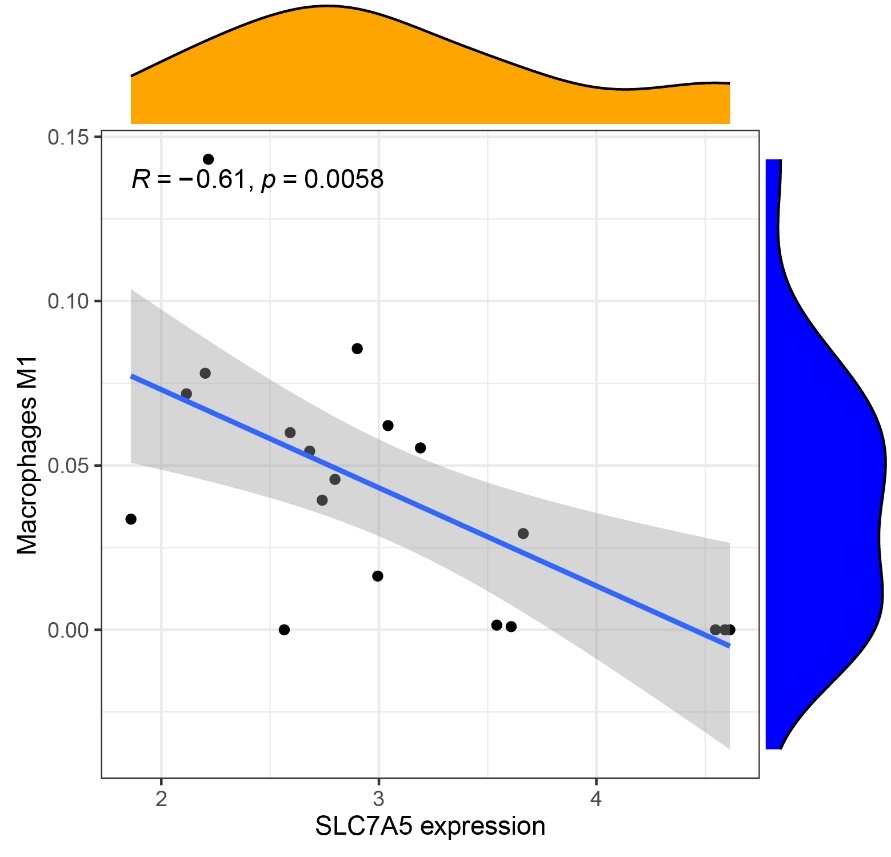


Supplementary figure 4: Correlation trend between *SLC7A5* and M1 macrophages


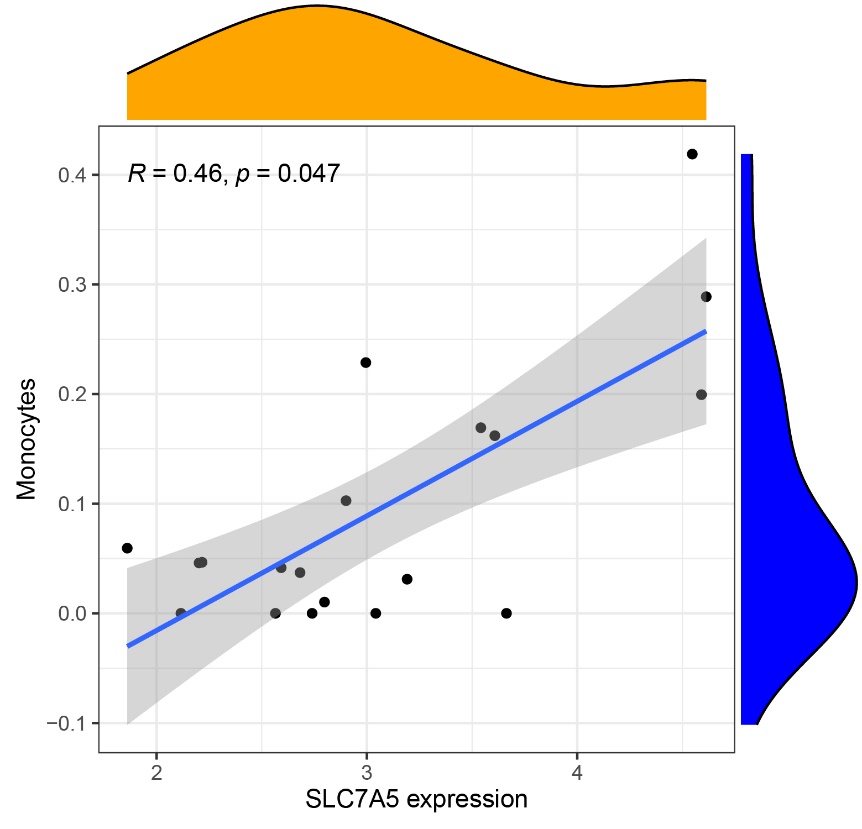


Supplementary figure 5: Correlation trend between *SLC7A5* and monocytes


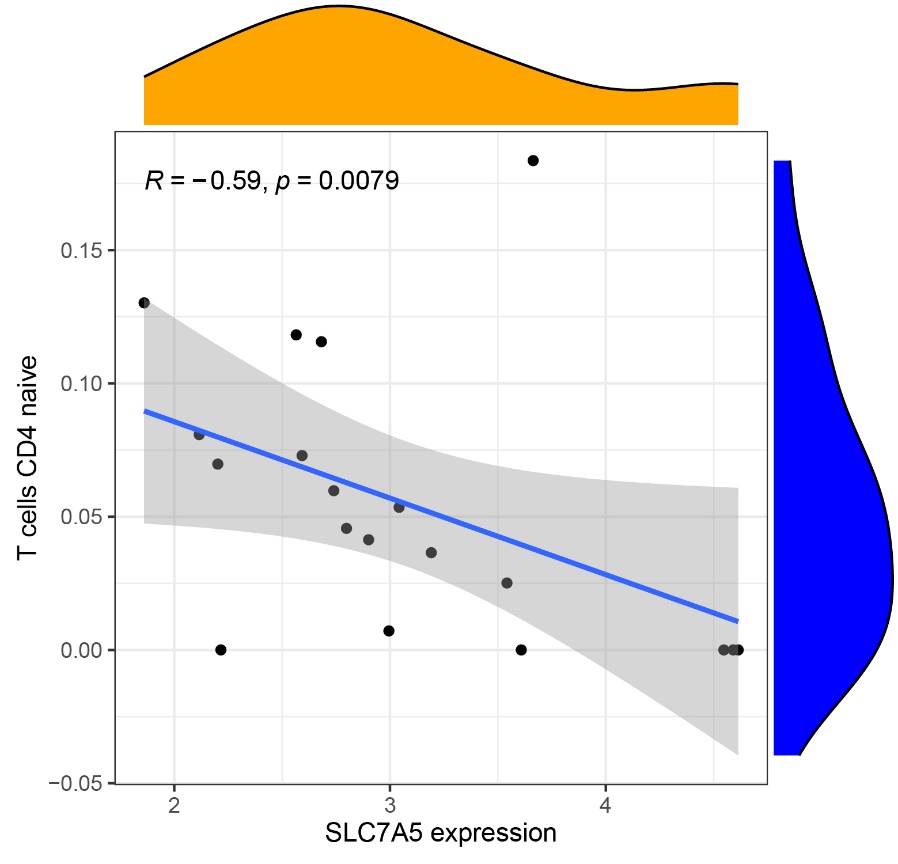


Supplementary figure 6: Correlation trend between *SLC7A5* and naive CD4 T cells


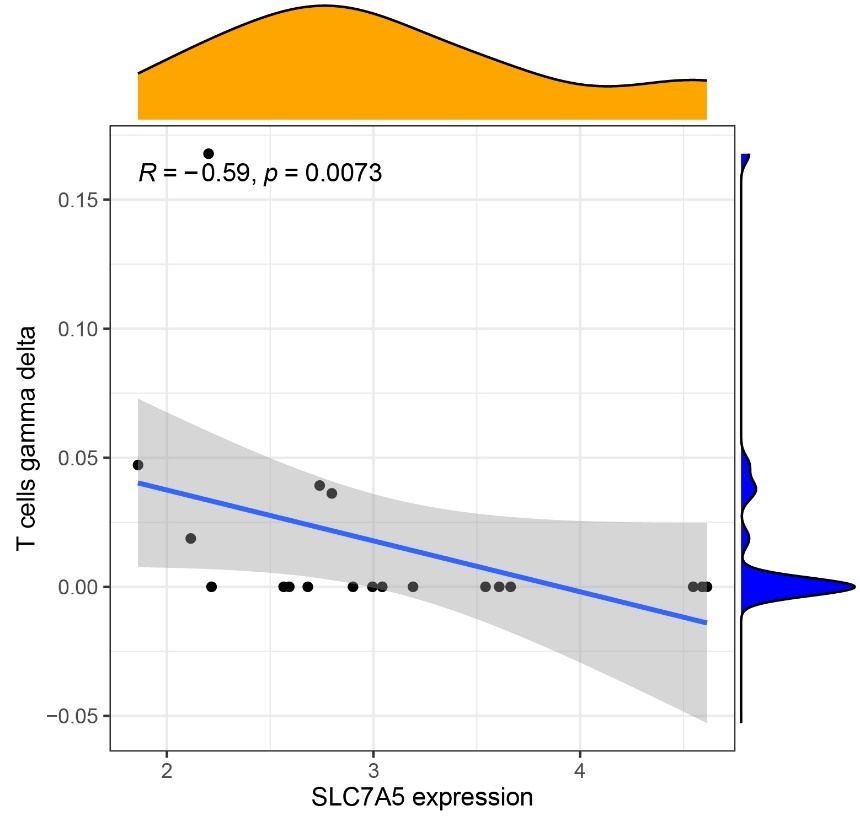


Supplementary figure 7: Correlation trend between *SLC7A5* and gamma delta T cells


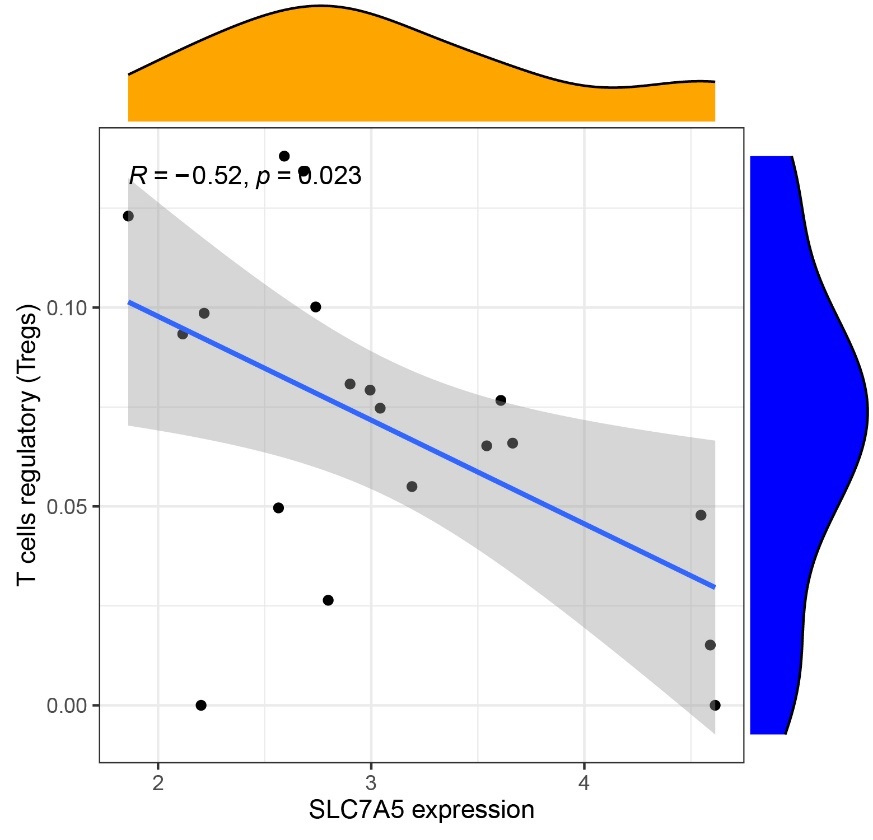


Supplementary figure 8: Correlation trend between *SLC7A5* and regulatory T cells (Tregs)


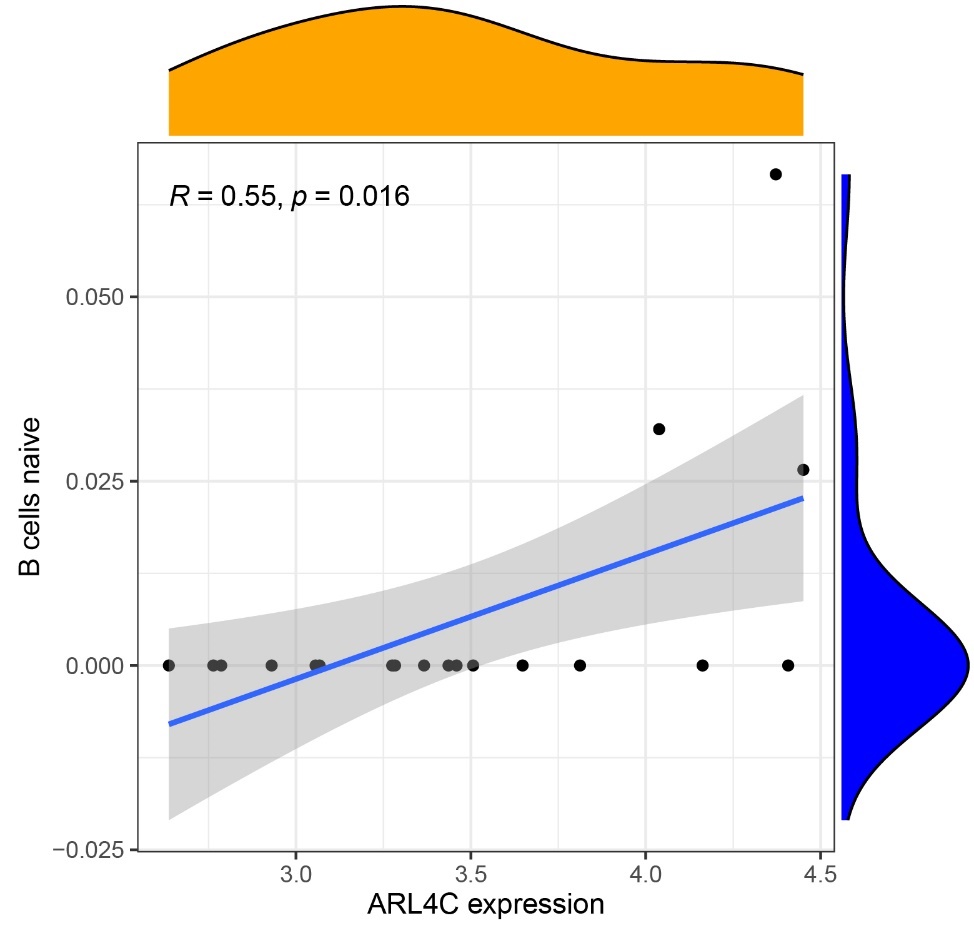


Supplementary figure 9: Correlation trend between *ARL4C* and naïve B cells
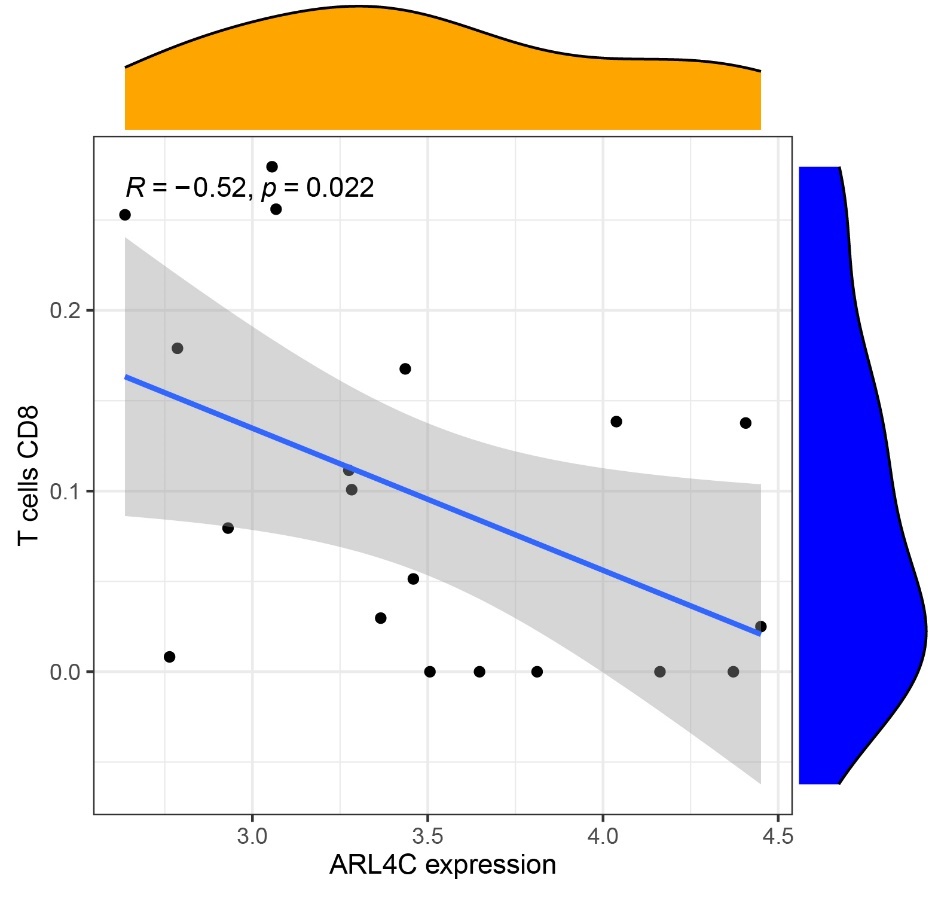


Supplementary figure 10: Correlation trend between *ARL4C* and CD8 T cells


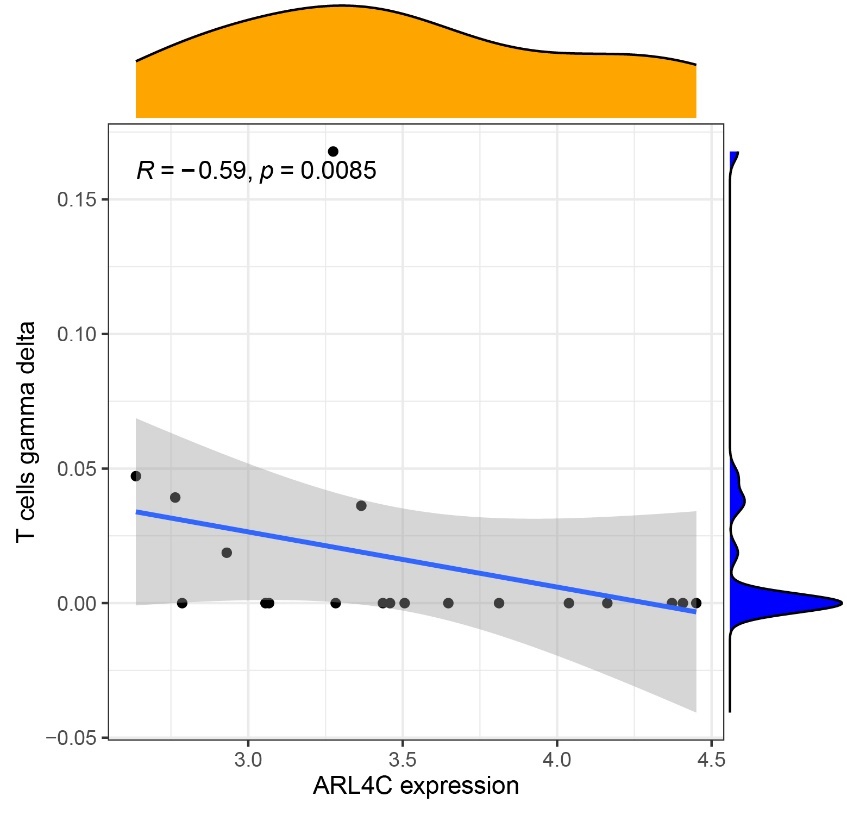


Supplementary figure 11: Correlation trend between *ARL4C* and gamma delta T cells


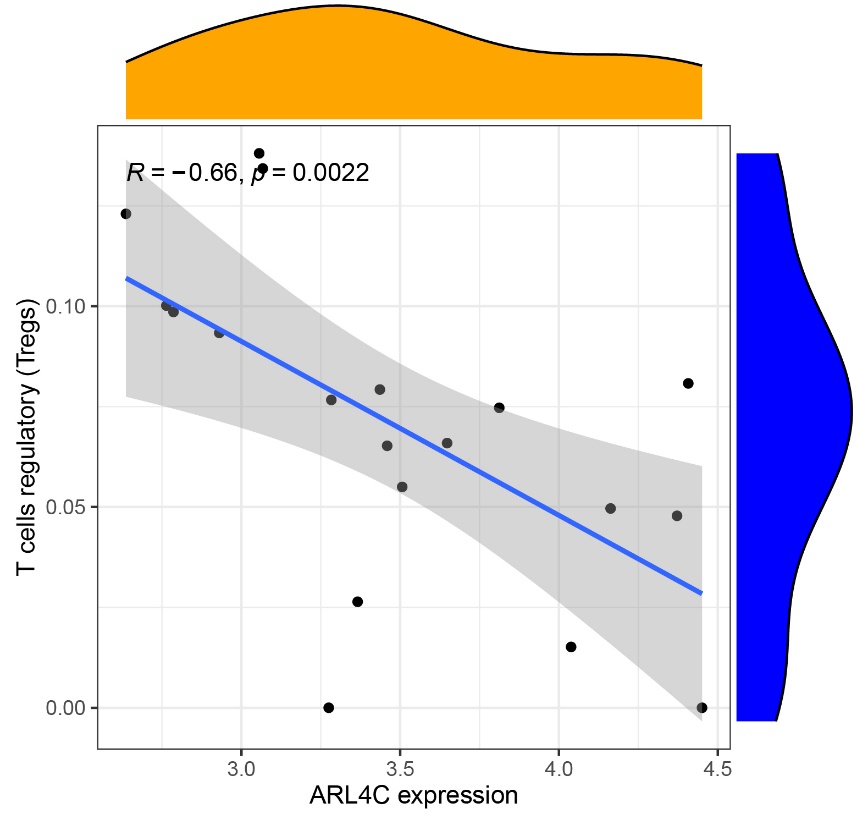


Supplementary figure 12: Correlation trend between *ARL4C* and regulatory T cells (Tregs)


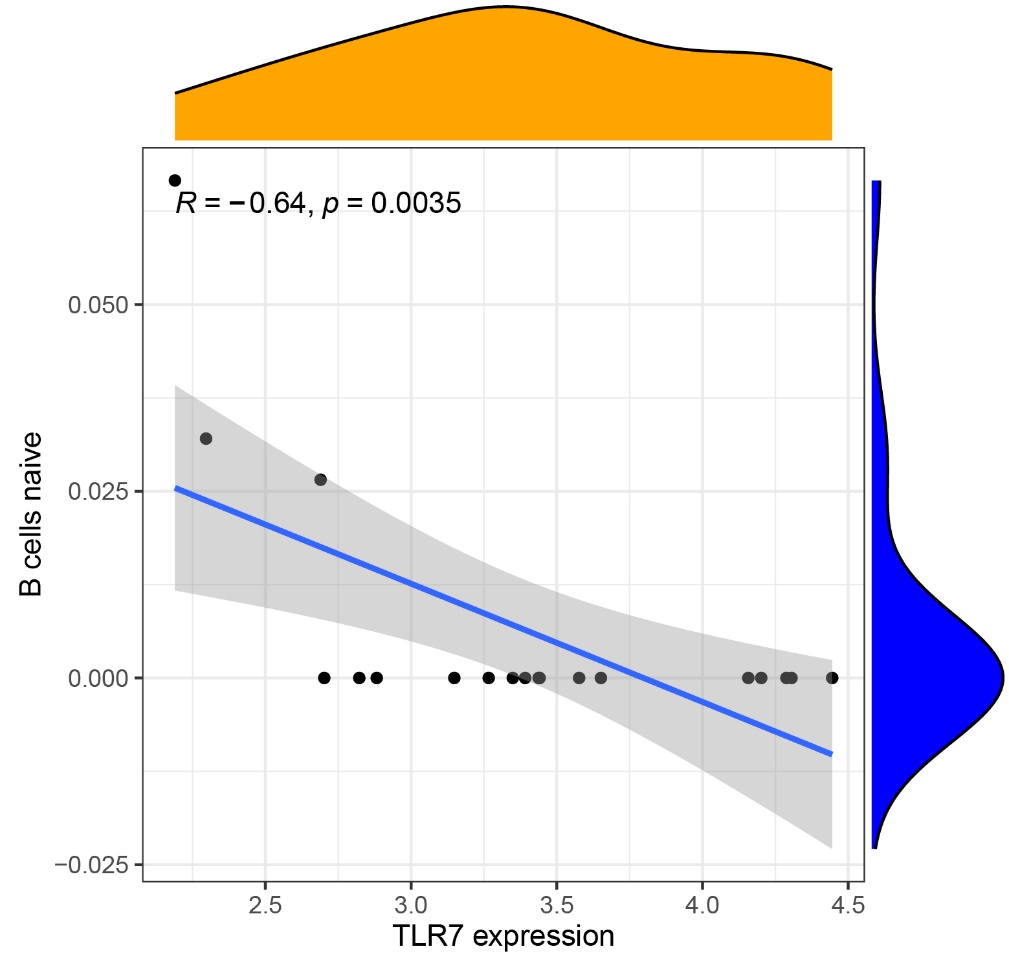


Supplementary figure 13: Correlation trend between *TLR7* and naïve B cells
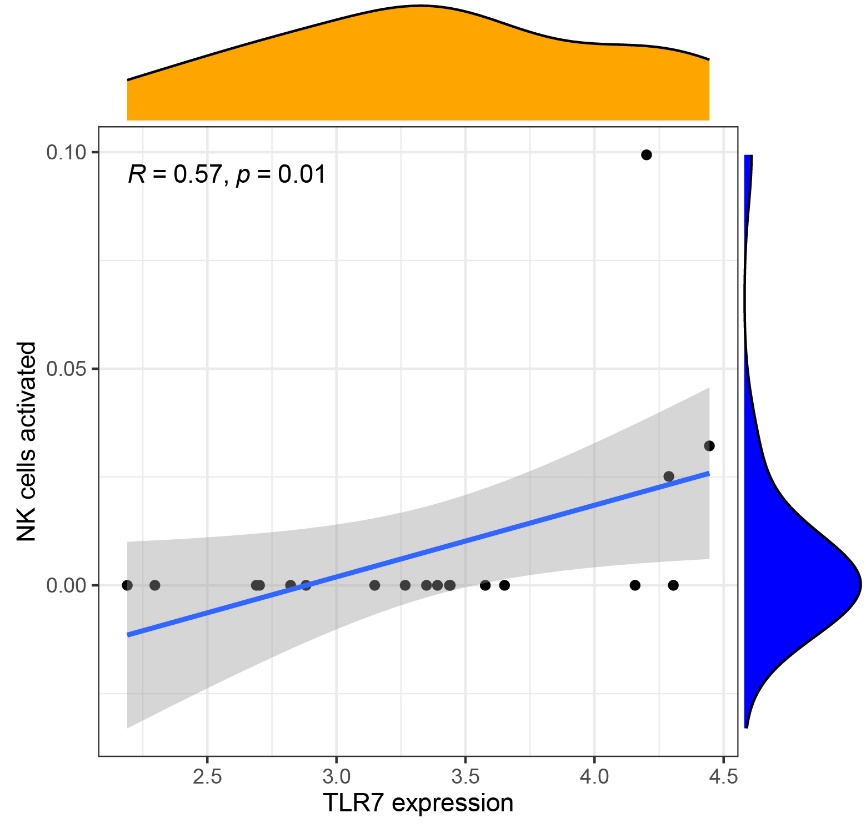


Supplementary figure 14: Correlation trend between *TLR7* and activated NK cells


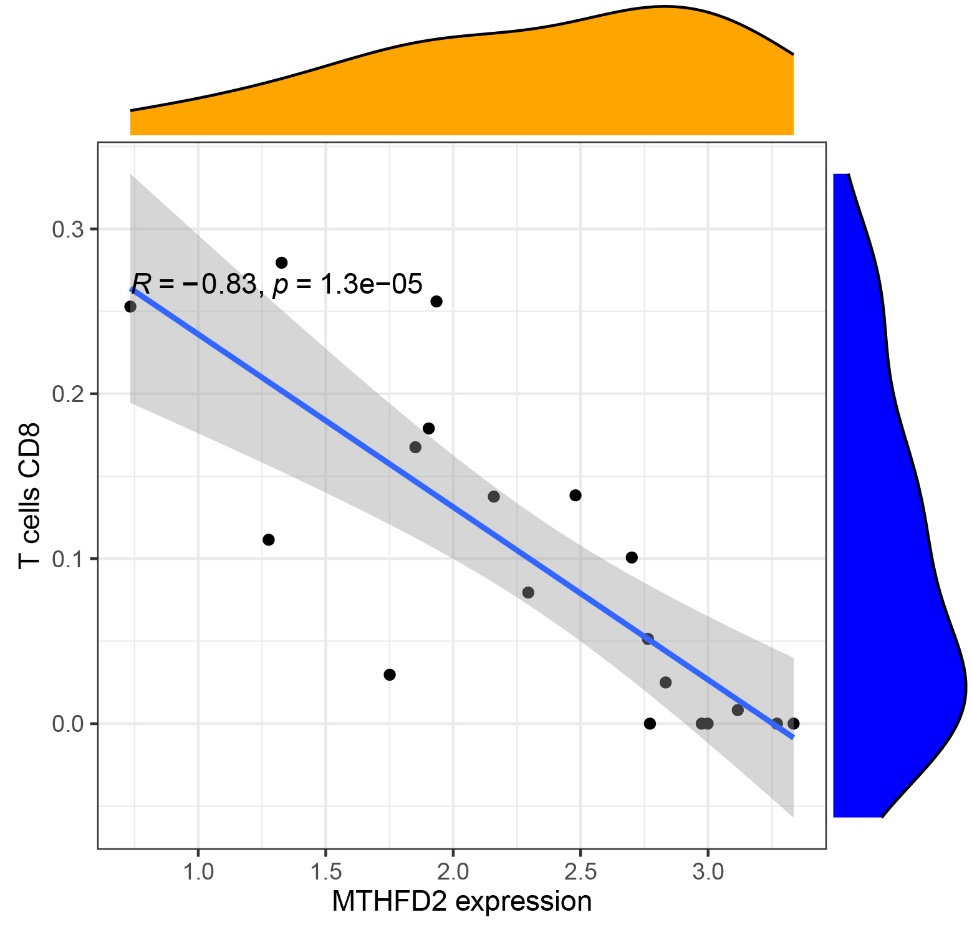


Supplementary figure 15: Correlation trend between *MTHFD2* and CD8 T cells
